# Aligning values and outcomes in priority–setting for health

**DOI:** 10.7189/jogh.06.020308

**Published:** 2016-12

**Authors:** Gabriel Seidman, Rifat Atun

**Affiliations:** Department of Global Health and Population, Harvard TH Chan School of Public Health, Harvard University, Boston MA, USA

Numerous factors and competing interests shape policymaking and budget allocation for health and health systems. In particular, the values and outcomes prioritized by policymakers have important implications for health spending and the impacts they have on populations and countries. Based on Harvard’s Ministerial Leadership Program, this article provides an overarching and integrative framework that policymakers can use to explicitly consider the priorities shaping their decisions, the outcomes that result from their decisions, and processes for making these decisions. The framework includes four key questions: 1) What values underlie the government’s priorities for the country? 2) Based on these values, what goals for the health care system does the government hope to achieve? 3) Based on these goals, where should the government allocate its financial resources for health? 4) How should the government allocate its financial resources for health? The framework also takes into consideration health system, economic, and political outcomes that result from budget allocations.

Investments in health and health systems can create value in two distinct but related ways: by generating “value for money” and “value for many” [1]. Policymakers can prioritize budgets to improve efficiency and effectiveness of health expenditures, thereby generating value for money, and target investments to improve equity and responsiveness to users’ needs, thereby achieving value for many.

The size and allocation of the health budget directly and indirectly impact population health. Low– and middle–income countries (LMICs) with similar per capita GDP, health expenditures as a proportion of GDP, and per person health expenditure have different outcomes. Investing in health can also bring economic benefits for countries [2–5] and political benefits for policymakers who choose to prioritize health (in countries where citizens have electoral power).

This article presents a framework (**Figure 1**) used at Harvard University’s Ministerial Leadership Program to introduce the roles that values and goals can play in prioritizing health programs and budgets. Based on our experience presenting this framework to several dozen Ministers of Health and Ministers of Finance, we believe it can serve two purposes. First, it can help policymakers explicitly articulate the values and principles that influence their decisions. Values and beliefs already influence decision–making; as discussed later, policymakers should make these values public and transparent to other decision–makers and the public in order to legitimize their decisions and to invite productive debate. Second, the framework explicitly links the way that different values can impact a policymakers’ priorities, which in turn can lead to different health, economic, and political outcomes. In line with the principles of systems thinking, we believe that by explicitly stating the “mental models” which guide decision–making, individuals and groups can better test their logic *ex ante* and evaluate the impact of their decisions *post hoc*.

**Figure 1:**
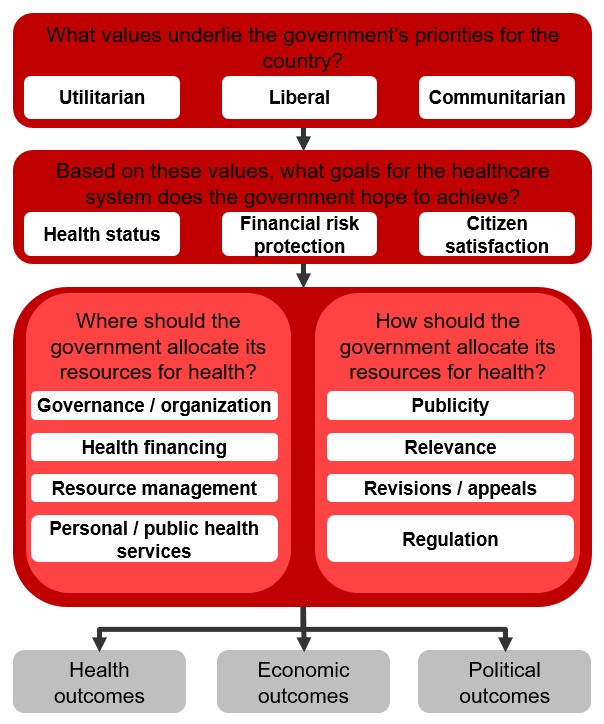
Framework for aligning values and outcomes when setting priorities for health.

The framework includes four guiding questions:

1. What values underlie the government’s priorities for the country?
2. Based on these values, what goals for the healthcare system does the government hope to achieve?
3. Based on these goals, where should the government allocate its financial resources for health?
4. How should the government allocate its financial resources for health?

These questions have direct relevance for all policymakers whose decisions impact health.

## 1. WHAT VALUES UNDERLIE THE GOVERNMENT’S PRIORITIES?

Although a broad range of values can drive the government’s approach to resource allocation, these value sets generally fall into three broad categories: utilitarian, liberal, and communitarian [2,6].

Utilitarians typically focus on the value, or utility, that a decision will have. Utilitarians generally believe “the ends justify the means” (assuming “the means” involve ethical and legal decisions). Policy tools such as cost–effectiveness and cost–benefit analysis reflect utilitarian concerns of generating the greatest benefits (utility) using the fewest possible resources. Utilitarians differ in how they choose to measure total utility. Subjective utilitarians argue that individuals must judge happiness for themselves. Objective utilitarians argue that individuals’ choices are not always rational and that allocating resources to maximize objective measures of well–being (eg, Disability–Adjusted Life Years [DALYs] and Quality–Adjusted Life Years [QALYs]) will have the greatest benefit.

Liberals take a rights–based approach to allocation of health resources. Liberals believe that humans have the capacity and obligation to display mutual respect, and this respect endows individuals with rights. Some liberals, known as libertarians, focus on negative rights, which guarantee individual freedom. Libertarians might focus on the rights of the individual to buy health insurance or choose their physician. In contrast, egalitarian liberals emphasize positive rights, or a minimum level of resources and services, which guarantee an individual’s ability to exercise free choice. Egalitarian liberals tend to favor redistribution of resources to ensure the entire population has access to positive rights. However, egalitarian liberals differ in their views on whether individuals have a right to health services (ie, provision of and access to care) or health status (ie, attaining general well–being).

Communitarians do not focus on the level of the individual in assessing a policy, but on the level of the community or society. They evaluate the merit of a policy based on whether it adheres to a community’s value set and whether the policy promotes a society consistent with those values. Communitarians would typically oppose a policy which achieves positive health outcomes using an intervention that defies local norms or values. Communitarians fall into two broad categories: those who believe in a single set of values which would promote a better society (universal communitarians), and those who argue that each society should set its own values based on context–specific factors (relativist communitarians).

These value sets are not mutually exclusive. Policymakers might include both a utilitarian and communitarian perspective in an analysis if they prioritize health interventions based on objective utility but exclude any that defy local norms. Further, governments can modify their ethical values as they learn more about a population’s needs and their ability to meet those needs. However, it is important to maintain adequate “coherence and explicitness” when articulating one’s values to create transparency for the population [2].

## 2. BASED ON THESE VALUES, WHAT GOALS FOR THE HEALTH CARE SYSTEM DOES THE GOVERNMENT HOPE TO ACHIEVE?

Policymakers must consider which outputs and outcomes to prioritize when allocating resources for health. In this context, *outputs* refers to how well the health system performs its functions, whereas *outcomes* refer to the ultimate goals of the health system. In many cases, strong delivery of health systems outputs is necessary but not sufficient for strong health system outcomes.

A policymaker needs to balance four health system outputs [7]:

- **Equity** refers to the differences in how a policy affects different people. “Vertical equity” evaluates differential impact across different populations, whereas “horizontal equity” evaluates whether the policy treats individuals with the same status the same [2].
- **Efficiency** has many definitions in the fields of policy analysis. For the purposes of health systems analysis, we draw on economic definition of technical efficiency, in which society is producing the most goods and services for the least cost [2].
- **Effectiveness** refers to whether interventions are evidence–based and safe [7]. In other words, an effective intervention will achieve the desired health outcomes.
- **Responsiveness** refers to whether the health system meets the public’s legitimate non–medical expectations. Responsiveness is a highly subjective measure and depends on the perceptions among citizens of a health system’s functioning [8].

Policymakers’ values will influence which health system outputs they prioritize. For example, pure utilitarians will likely care most about efficiency and effectiveness, and they will less likely prioritize equity. They might also disregard the importance of responsiveness as an objective, unless they believe that a health system’s responsiveness generates value for the population. Liberals, who focus on individuals’ rights, will prioritize equity and responsiveness of the system, with libertarians emphasizing the importance of responsiveness (eg, choice of health service providers) and egalitarian liberals emphasizing equity in access to positive rights (eg, basic health services and medicines). Communitarians, who emphasize society’s values, will prioritize the objectives most relevant for achieving the best possible society. Accordingly, they will likely emphasize responsiveness and equity of the system at a societal level, although the emphasis could vary depending on the specific values of the society.

In addition to setting output objectives, policymakers must also pay attention to the health systems outcomes, or the overall goals, of a country’s health system [2,7]:

- **Health status** refers to the health of a population. Measurements of population health status include life expectancy, burden of disease, mortality rates for specific groups, and disease prevalence.
- **Financial risk protection** refers to helping people avoid large and unpredictable payments for health, also known as catastrophic (or impoverishing) expenditures. Mechanisms to provide financial risk protection typically involve insurance schemes or tax–funded health systems.
- **Citizen satisfaction** refers to the degree with which users of the health system rate the system as satisfactory.

As with outputs, health systems outcomes derive directly from values. For example, objective utilitarians might concern themselves most with the population’s average health status, whereas egalitarian liberals might focus most on the distribution or range of health statuses in the population (as a measure of equity). Egalitarian liberals will also emphasize the importance of financial risk protection as a means for ensuring economic opportunities for all. Subjective utilitarians might place a high value on citizen satisfaction, as would libertarians (in the sense that satisfaction relates to individual choice).

## 3. BASED ON THESE GOALS, WHERE SHOULD THE GOVERNMENT ALLOCATE ITS FINANCIAL RESOURCES FOR HEALTH?

Once the government has identified its objectives for the outputs and defined its goals for the health system, it can invest in specific programs or interventions accordingly. A health system has four main functions which a government can prioritize for investment [7]:

- **Governance and organization** encompasses the institutions involved in delivering products and services to citizens such as hospitals and primary care clinics [9]. Investments in this function include improving accountability or transparency of decision–making, updating management policies and processes at the programmatic level, or changing the system’s referral network.
- **Health financing** involves mobilizing, pooling, and allocating financial resources. A government could choose to invest in health financing by creating a new insurance scheme, expanding coverage of existing insurance to new patient populations, or by expanding the range of services covered under existing schemes.
- **Resource management** entails overseeing the inputs, such as human resources and labor, pharmaceuticals, and medical technologies that produce personal or public health services. The government can invest in the management of resources by purchasing these resources (eg, procuring medicines), improving systems that oversee and deliver resources (eg, budgeting tools, supply chain management), or by investing in infrastructure and human resources to strengthen the health system [10].
- **Personal and public health services** refer to the activities involved in delivering care to patients. Strong health systems enable delivery of these services. Governments also invest in specific services that generate value, such as by investing in primary health care delivery. Several investment cases have been made for disease–specific “good buys” such as those identified by the Lancet Commission on Investing in Health [11]; UNAIDS HIV Investment Framework [12]; STOP TB Strategy [13]; the Global Strategy for Women's and Children's Health spearheaded by the UN Secretary General [14]; interventions identified in the Global Malaria Action Plan [15], and the Package of Essential Noncommunicable Disease Interventions (also known as WHO–PEN) [16].
  Values will influence how policymakers invest across these four functions. For example, a utilitarian might focus on improving resource management to reduce wastage in the system and improve efficiency. Utilitarians might also focus on the “good buy” interventions described above and choose to invest in those that improve population health for the least cost. By contrast, egalitarian liberals might focus on ensuring equitable access to health insurance and effective health services, especially for marginalized patients such as poor and rural populations, even if these programs are more expensive. Communitarians will focus on implementing these functions to coincide with their society’s values. For example, a society that emphasizes individual responsibility for health might de–prioritize social support for accessing services, while a society that emphasizes the community’s role in promoting health might implement a social health insurance scheme or mobilize the community to raise awareness about disease prevention.

## 4. HOW SHOULD THE GOVERNMENT ALLOCATE ITS FINANCIAL RESOURCES FOR HEALTH?

There is no formula for determining which health interventions or areas to prioritize, and limiting analyses to comparisons of cost–effectiveness is insufficient for policymaking.

Without universal consensus on the principles for prioritization, governments need to adopt an approach to allocate resources and justify their policies. Accordingly, ethicists have proposed a framework known as “accountability for reasonableness” (A4R) to guide this decision–making process. A4R, a process grounded in democratic principles aimed at legitimizing decision–making among “ ‘fair–minded’ people who seek mutually justifiable terms of cooperation,” has four conditions [17]:

1. Publicity: Decisions that establish priorities in meeting health needs and their rationales must be publicly accessible.
2. Relevance: Policymakers should provide reasonable rationales which appeal to evidence, reasons, and principles accepted as relevant by fair–minded people when justifying their decisions. Rationales should be relevant for a broad range of stakeholders in decision–making.
3. Revision and appeals: There must be mechanisms for challenge and dispute and, more broadly, opportunities for revision and improvement of policies in light of new evidence or arguments.
4. Regulative: There must be public regulation of the process to ensure that conditions 1, 2, and 3 are met.

A4R does not identify the priorities for government investments; it establishes a transparent process for publicly and legitimately determining these priorities in order to guide investment decisions. These principles have relevance for policymakers and societies that subscribe to all value sets. Indeed, A4R does not promote a specific value set, but rather advocates for explicitly articulating and linking values and principles to decisions and outcomes, which our framework can help put into practice.

The principles of A4R have influenced health priority–setting in several places: UK, where the National Institute for Health and Clinical Excellence (NICE) takes social value judgments into account when recommending coverage for new treatments [18]; Mexico, where decisions about which diseases the public catastrophic insurance should cover involve working groups that evaluate clinical, economic, ethical, and social considerations [19]; and Oregon, where, in 2008, a Health Fund Board made a plan to insure all legal residents of the state involving a wide group of stakeholders and extremely transparent decision–making / information–sharing [20].

## OUTCOMES FROM HEALTH SPENDING

The decisions described above can have at least three sets of outcomes.

### Health system outcomes

Changes in government health spending can directly impact cause–specific mortality. For example, in low–income countries a 1% decrease in government health spending is associated with an increase of 18 neonatal deaths for every 100 000 live births and 98 deaths before the age of five [21]. From 1999–2004, a 10% increase in per capita total health expenditure was associated with a 22% reduction in infant mortality rate and 10% increase in per capita public health expenditure was associated with a 21% reduction in infant mortality rate [22]. Globally, a 1% increase in government health spending is also associated with a significant decrease in cerebrovascular deaths [23].

However, simply increasing government (or any) spending on health will not necessarily improve health outcomes, especially if funds are not spent efficiently. Evidence suggests that increasing the efficiency of government health spending, without increasing total budget expenditure, could improve population health outcomes [5]. Increasing health spending efficiency among nations below the regional average to the regional average would result in an increase in health–adjusted life expectancy (HALE) by 1.5 years in Africa, 1 year in Asia/Pacific, and 1.3 years in Middle East / Central Asia. In the most extreme example, increasing health spending efficiency in Sierra Leone to the average for Africa could improve HALE by 5.3 years.

Achieving the health systems goal of financial risk protection through universal health coverage (UHC) can also improve population health status. Countries that currently do not have UHC can improve coverage either by increasing budget allocation to health, or by improving spending efficiency in order to redirect spending to UHC. Cross–country analysis of the influence of insurance coverage on health outcomes suggests that financial coverage has a causal influence on health, especially for low–income individuals, who gain better access to necessary care when they receive coverage [24]. Individual countries’ experiences implementing UHC, including Thailand, Turkey, and several countries in Latin America, supports this finding [7,25–27].

### Economic outcomes

Evidence strongly suggests that improved population health has positive economic impacts for a country. Achieving better population health provides a sound “return on investment” in the form of stronger economic output and growth. Evidence for the linkage between health and economic output exists at both the microeconomic and macroeconomic levels.

At the microeconomic level, better health can improve the financial prospects of individuals and households [3]. Malnutrition, frequent illness, and unstimulating home environments can limit the physical and cognitive development of children. Conversely, proper nutrition and health supports adequate physical development and school performance [3]. Interventions targeting specific diseases, such as deworming, nutrition supplements, and malaria prevention can lead to improved education or income outcomes for individuals [4]. Among working individuals, illness can negatively impact income due to impoverishing health expenditures, reduced education opportunities, decreased productivity at work, long–term separation from the work force, and disengagement from other economic activities.

Macroeconomic evidence also supports the idea that investing in health generates positive economic returns [4]. First, *ceteris paribus*, a healthy workforce will have higher labor productivity than an unhealthy workforce due to increased energy and reduced illness–related absenteeism. Second, a healthy population has increased educational opportunities, and education levels have a direct impact on a country’s income growth. Third, populations with high life expectancies tend to save more for the future and likely will have more working years. These increased savings can lead to increased investable capital, an important driver of growth. Fourth, health investments that change mortality and fertility can lead to a “demographic dividend,” in which the ratio of working–age to non–working–age people in the country increases and productive capacity increases on a per capita basis. (This demographic dividend accounts for up to one–third of the economic boom that many East Asian countries experienced between 1965 and 1990.)

### Political outcomes

Formulating health policy and allocating resources to health depends on and also impacts a country’s politics. For example, the transition toward universal health coverage (UHC) has had distinct positive political benefits in many countries [28]. In addition, health policy in countries such as Turkey, the UK and Brazil has influenced the political landscape and political outcomes.

In Turkey, after a regime change in 2002, the government implemented a Health Transformation Program (HTP) with significant commitment from political leadership. This transformation led to increased levels of public satisfaction with the government [7] and influenced voter intentions in favor of the government [29].

**Figure:**
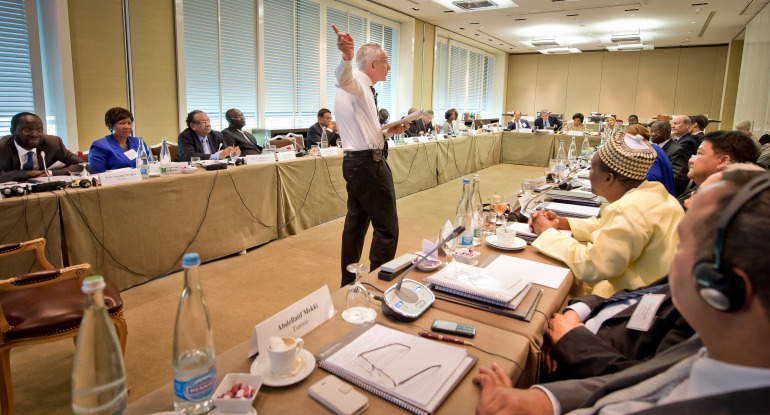
Photo: Ministers of Health participating in a Roundtable Meeting hosted by the Harvard Ministerial Leadership Program. Photo courtesy of the Harvard Ministerial Program.

After the re–democratization of the Brazilian government, the 1988 constitution formally defined health as a “citizen’s right and obligation of the state” and established the Unified Health System (SUS), which sought to unify the fragmented care delivery network into a national health system under the MoH [30]. Today, 75% of Brazil’s population, or 195 million people, receive services and coverage from SUS [31].

In the UK, the National Health Service (NHS) receives broad public support, with 89% of the public agreeing with the idea of a tax–funded national health system, which is managed by the government. However, projections show that by 2030, the NHS will have a £65 billion funding gap. Therefore, UK policymakers will have to balance the competing health, financial, and social demands placed on the NHS in order to maintain its relevance going forward.

## FRAMEWORK LIMITATIONS

Although we believe that this framework can help policymakers make their values more explicit and link values to decisions and outcomes, it has several limitations. First, it presents decision–making as a linear process, whereas decision–making occurs in the context of complicated processes subject to outside forces. Second, the framework does not incorporate decision analysis tools into its approach and cannot and cannot provide a clear–cut answer when making a tradeoff between two different investments. We believe that linking analysis of value–based decision–making with quantitative decision tools is an important next step for study. Finally, because this framework focuses by design on the level of health systems, clinicians cannot use it to make individual care decisions.

## CONCLUSION

This article introduces a framework for policymakers to consider how their values influence priority–setting for health and the impacts that these priorities can have on health systems, economic, and political outcomes. By clearly articulating values and priorities, policymakers can develop a transparent and deliberative process to better discuss and engage their constituents in health systems decisions and to set priorities. These priorities, in turn, can generate value for money by improving efficiency and effectiveness of budget allocation decisions and value for many by enhancing equity and responsiveness in the health system.

## References

[1] Atun R (2015). The National Health Service: value for money, value for many.. Lancet.

[2] Roberts M, Hsiao W, Berman P, Reich M. Getting health reform right: a guide to improving performance and equity. Oxford: Oxford University Press; 2008.

[3] Jack W, Lewis M. Health investments and economic growth: macroeconomic evidence and microeconomic foundations. World Bank Policy Research Working Paper Series. Available: https://openknowledge.worldbank.org/handle/10986/4072. Accessed: 10 October 2016.

[4] Bloom D, Fink G. The economic case for devoting public resources to health. Manson’s Tropical Diseases. Amsterdam: Elsevier Ltd; 2013.

[5] Grigoli F, Kapsoli J. International Monetary Fund. Waste not, want not: the efficiency of health expenditure in emerging and developing economies. 2013. Available: https://www.imf.org/external/pubs/cat/longres.aspx?sk=40899.0. Accessed: 7 December 2015.

[6] Roberts MJ, Reich MR (2002). Ethical analysis in public health.. Lancet.

[7] Atun R, Aydın S, Chakraborty S, Sümer S, Aran M, Gürol I (2013). Universal health coverage in Turkey: enhancement of equity.. Lancet.

[8] World Health Organization. The World Health Report: 2000: health systems: improving performance. 2000. Available: http://www.who.int/whr/2000/en/. Accessed: 7 December 2015.

[9] Hsaio WC (1995). Abnormal economics in the health sector.. Health Policy.

[10] Shakarishvili G, Lansang MA, Mitta V, Bornemisza O, Blakley M, Kley N (2011). Health systems strengthening: a common classification and framework for investment analysis.. Health Policy Plan.

[11] Jamison DT, Summers LH, Alleyne G, Arrow KJ, Berkley S, Binagwaho A (2013). Global health 2035: a world converging within a generation.. Lancet.

[12] Schwartländer B, Stover J, Hallett T, Atun R, Avila C, Gouws E (2011). Towards an improved investment approach for an effective response to HIV/AIDS.. Lancet.

[13] Raviglione MC, Uplekar MW (2006). WHO’s new Stop TB strategy.. Lancet.

[14] United Nations Secretary General. Global strategy for women’s and children’s health. 2010. Available: http://www.who.int/pmnch/activities/advocacy/fulldocument_globalstrategy/en/. Accessed: 7 April 2016.

[15] Roll Back Malaria. The global malaria action plan. 2008. Available: http://www.rollbackmalaria.org/microsites/gmap/0-5.pdf. Accessed: 7 April 2016.

[16] World Health Organization. Package of essential noncommunicable (PEN) disease interventions for primary health care in low-resource settings. 2010. Available: http://www.who.int/nmh/publications/essential_ncd_interventions_lr_settings.pdf. Accessed: 7 December 2015.

[17] Gruskin S, Daniels N (2008). Process is the point: justice and human rights: priority setting and fair deliberative process.. Am J Public Health.

[18] Daniels N, Sabin JE (2008). Accountability for reasonableness: an update. BMJ.

[19] Daniels N. Accountability for reasonableness in developing countries. In Just health: meeting health needs fairly. Cambridge: Cambridge University Press; 2007.

[20] Daniels N (1999). Decisions about access to health care and accountability for reasonableness.. J Urban Health.

[21] Maruthappu M, Ng KY, Williams C, Atun R, Zeltner T (2015). Government health care spending and child mortality.. Pediatrics.

[22] Anyanwu JC, Erhijakpor AE (2009). Health expenditures and health outcomes in Africa.. Afr Dev Rev.

[23] Maruthappu M, Shalhoub J, Tariq Z, Williams C, Atun R, Davies AH (2015). Unemployment, government healthcare spending, and cerebrovascular mortality, worldwide 1981–2009: an ecological study.. Int J Stroke.

[24] Moreno-Serra R, Smith PC (2012). Does progress towards universal health coverage improve population health?. Lancet.

[25] Hanvoravongchai P. Health financing reform in Thailand: toward universal coverage under fiscal constraints. Universal Health Coverage Studies Series 2013. Available: https://openknowledge.worldbank.org/bitstream/handle/10986/13297/75000.pdf. Accessed: 7 April 2016.

[26] Limwattananon S, Tangcharoensathien V, Tisayaticom K, Boonyapaisarncharoen T, Prakongsai P (2012). Why has the Universal Coverage Scheme in Thailand achieved a pro-poor public subsidy for health care?. BMC Public Health.

[27] Atun R, de Andrade LO, Almeida G, Cotlear D, Dmytraczenko T, Frenz P (2015). Health-system reform and universal health coverage in Latin America.. Lancet.

[28] Yates R, Humphreys G. Arguing for universal health coverage. 2013. Available: http://www.who.int/health_financing/UHC_ENvs_BD.PDF. Accessed: 7 April 2016.

[29] Esen B. Myths and facts about Turkey's welfare regime. 2014. Available: http://www.dailysabah.com/opinion/2014/08/26/myths-and-facts-about-turkeys-welfare-regime. Accessed: 7 April 2016.

[30] Couttolenc B, Dmytraczenko T. The World Bank. Brazil’s primary care strategy. 2013. Available: http://documents.worldbank.org/curated/en/881491468020373837/Brazils-primary-care-strategy. Accessed: 7 December 2015.

[31] Kepp M (2014). Upcoming election could rekindle health debate in Brazil.. Lancet.

